# *Aspergillus flavus* infection induces transcriptional and physical changes in developing maize kernels

**DOI:** 10.3389/fmicb.2014.00384

**Published:** 2014-07-31

**Authors:** Andrea L. Dolezal, Xiaomei Shu, Gregory R. OBrian, Dahlia M. Nielsen, Charles P. Woloshuk, Rebecca S. Boston, Gary A. Payne

**Affiliations:** ^1^Monsanto CompanyWaterman, IL, USA; ^2^Department of Plant Pathology, North Carolina State UniversityRaleigh, NC, USA; ^3^Department of Genetics, North Carolina State UniversityRaleigh, NC, USA; ^4^Department of Botany and Plant Pathology, Purdue UniversityWest Lafayette, IN, USA; ^5^Department of Plant and Microbiological Sciences, North Carolina State UniversityRaleigh, NC, USA

**Keywords:** *Aspergillus flavus*, maize, transcription, genetic, aflatoxins, pathogenesis

## Abstract

Maize kernels are susceptible to infection by the opportunistic pathogen *Aspergillus flavus*. Infection results in reduction of grain quality and contamination of kernels with the highly carcinogenic mycotoxin, aflatoxin. To understanding host response to infection by the fungus, transcription of approximately 9000 maize genes were monitored during the host-pathogen interaction with a custom designed Affymetrix GeneChip® DNA array. More than 4000 maize genes were found differentially expressed at a FDR of 0.05. This included the up regulation of defense related genes and signaling pathways. Transcriptional changes also were observed in primary metabolism genes. Starch biosynthetic genes were down regulated during infection, while genes encoding maize hydrolytic enzymes, presumably involved in the degradation of host reserves, were up regulated. These data indicate that infection of the maize kernel by *A. flavus* induced metabolic changes in the kernel, including the production of a defense response, as well as a disruption in kernel development.

## Introduction

*Aspergillus flavus* is an opportunistic fungal pathogen that infects developing maize kernels, attacking plants that are weakened by environmental stresses such as drought and heat. Disease reduces grain quality and contaminates the kernel with the carcinogenic mycotoxin aflatoxin (Scheidegger and Payne, 2003; Payne and Yu, 2010; Dolezal et al., 2013; Hruska et al., 2013; Kew, 2013). The development of resistant maize lines has proven difficult although there is evidence for sources of resistance (Brown et al., 1999; Windham and Williams, 2002; Mylroie et al., 2013; Warburton et al., 2013; Mideros et al., 2014). The lack of reliable resistance phenotyping markers, the inconsistency of disease development each year, and an insufficient understanding of host resistance mechanisms, all have made the selection of resistance difficult.

Advances in technology, such as microarrays, have enabled researches the ability to monitor transcription on a genome-wide level and provided a better understanding of how organisms respond to their environment on a cellular level. Studies investigating plant gene expression during pathogen attack have found the defense response goes beyond PR-proteins and involves transcription changes in both primary and secondary plant metabolic pathways and detoxification pathways (Boddu et al., 2007; Doehlemann et al., 2008; Alessandra et al., 2010). Phytohormones like salicyclic acid (SA), jasmonic acid (JA), ethylene (ET) have long been known to be an integral part of the defense response (Glazebrook, 2005; Jones and Dangl, 2006; Robert-Seilaniantz et al., 2011). Yet carbohydrate metabolism pathways, though not typically associated with resistance, may be an important component of the plant defense response including in maize (Berger et al., 2007; Bolton, 2009). Higher maize stalk carbohydrate levels have been associated with increased resistance to stalk infecting fungi, many of which are also capable of infecting the ear and kernel (Dodd, 1980).

Transcriptional changes of maize kernels during infection by *A. flavus* have been studied using microarrays (Luo et al., 2011; Kelley et al., 2012) and qPCR (Jiang et al., 2011). Kelley et al. (2012) compared maize varieties that were either susceptible or resistant to aflatoxin accumulation. They found 16 genes highly expressed in the resistant variety and 15 in the susceptible variety and concluded that multiple mechanisms are likely involved in resistance to aflatoxin accumulation. Jiang et al. (2011) reported higher levels of gene expression in stress related genes in resistant lines of maize. Luo et al. (2011) found that more maize genes were induced by *A. flavus* in susceptible kernels compared with resistant kernels. In all these studies, defense-related, and regulatory genes were associated with the response to *A. flavus*. To provide a clearer understanding of maize kernel resistance to *A. flavus* we monitored the transcriptional response of maize kernels during infection by *A. flavus* in the field using a custom DNA microarray. We report changes in expression of well-characterized defense signaling pathways and defense related genes as well as striking changes in expression of genes related to carbohydrate metabolism.

There are several stages in the infection process that host resistance could restrict fungal growth and aflatoxin contamination. Kernel infection with *A. flavus* begins through silk colonization. Conidia germinate and grow on senescing silks, moving down the silk channel to the developing kernels, which can take as little as 8 days (Marsh and Payne, 1984; Payne et al., 1988b). Subsequent steps in the infection process are less defined, but data suggest that *A. flavus* can attack kernels during their six stages (Ritchie et al., 1997) of their development: silking (R1), blister (R2), milk (R3), dough (R4), dent (R5), and physiological maturity (R6). Recently, Reese et al. (2011) inoculated detached kernels at stages R2–R5 in the lab and found that kernels at these four stages were susceptible to infection by *A. flavus*. Fungal infection has been observed in injured kernels as young as the milk (R3) stage (Taubenhaus, 1920; Anderson et al., 1975). These young kernels tend to accrue high concentrations of aflatoxin because of prolonged colonization by the pathogen (Lillehoj et al., 1980; Payne et al., 1988a). Infection in non-injured kernels in the field is thought to take place later, during the dent (R5) developmental stage just prior to physiological maturity (R6) (Koehler, 1942; Marsh and Payne, 1984; Payne et al., 1988a; Smart et al., 1990; Windham and Williams, 1998). Once inside, *A. flavus* preferentially colonizes the oil-rich germ tissue (Fennell et al., 1973; Jones et al., 1980; Smart et al., 1990; Keller et al., 1994). Fungal growth within endosperm tissue, more specifically the nutrient-rich starchy endosperm, has been observed, but there are discrepancies in the literature as to the extent of colonization (Lillehoj et al., 1976; Smart et al., 1990; Keller et al., 1994; Brown et al., 1995; Dolezal et al., 2013).

Our studies focused on the transcriptional response of developing kernels that were inoculated with *A. flavus* through a wound. We realize that this approach could overlook some resistance mechanisms, but it results in more consistent disease development. Resistance to infection of wounded kernels is also relevant as it mimics insect injury, which is important in the epidemiology of the disease. Furthermore, to capture the response in the different stages of kernel development, we evaluated *A. flavus* infection of four kernels stages, R2–R5. We also chose a specific time of 4 days after inoculation to examine gene expression based on previous histological studies by Dolezal et al. (2013) who showed that within 4 days after inoculation *A. flavus* mycelium reached the aleurone, endosperm, and germ tissue. Thus, sampling at 4 days allowed assessment of host response in several tissue types within the kernels.

## Experimental procedure

### Fungal strain and culture conditions *Aspergillus flavus*

NRRL 3357 was grown on potato dextrose agar (PDA) at 28°C for 7–10 days. Conidia were dislodge with 0.05% (v/v) Triton X-100 and diluted to a working solution of 1 × 10^6^ spores mL^−1^.

### Maize kernel inoculation and harvesting

Inbred maize genotype B73 was grown at the Central Crops Research Station in Clayton, NC. Ears were hand pollinated and the date recorded on the bag. Ears at the blister (R2), milk (R3), late milk (R3)–early dough (R4), dough (R4), and dent (R5) stages of development were either mock-inoculated or inoculated with *A. flavus* as outline in Dolezal et al. (2013). Briefly, ears selected for inoculation had the husk pulled back to exposed the developing kernels below. The protruding portion of the pins of the pinbar was dipped into the *A. flavus* conidial suspension and inserted into the crown of the kernel. The husk was repositioned and secured around the ear with a rubber band, and a paper bag placed over the inoculated ear. Ears inoculated at the blister (R2), milk (R3), dough (R4), and dent (R5) stages of development were removed from the plant 4 days after inoculation (dai), and the kernels flash frozen immediately after removal. Harvested kernels were stored at −80°C until RNA was extracted using the protocol outlined in Smith et al. (2008). Additional ears inoculated at the late milk (R3)–early dough (R4) stage of development were left in the field and picked at end of the growing season. Kernels adjacent to the pinbar-inoculated rows were harvested. Kernels on non-inoculated ears, pollinated the same day as the inoculated ears, were also collected and used as controls. Adjacent diseased kernels and control kernels were cut-in-half and visually compared to assess for physical changes in kernel structure resulting from *A. flavus* infection.

### Microarray processing and analysis

Custom-designed *A. flavus* Affymetrix GeneChip DNA microarrays were used to identify genes differentially expressed in maize during *A. flavus* kernel colonization. This multi-species array, in addition to being capable of monitoring genome-wide transcription of *A. flavus*, has close to 9000 probe sets representing maize genes. This pairing of *A. flavus* and maize genes onto a single array allowed for simultaneous detection of disease-associated transcript in the plant-pathogen interaction. The majority (83%) of maize genes selected for the array came from seed-specific cDNA libraries. The remaining genes were chosen based on recommendations from members of the maize community and prior association with disease resistance. The quality of RNA extracted from the mock-inoculated and *A. flavus*-inoculated kernels was assessed before processing. All array work was carried out at the Purdue Genomic Core Facility (http://www.genomics.purdue.edu) in West Lafayette, IN, and standard Affymetrix protocols were followed.

**Figure 1:**
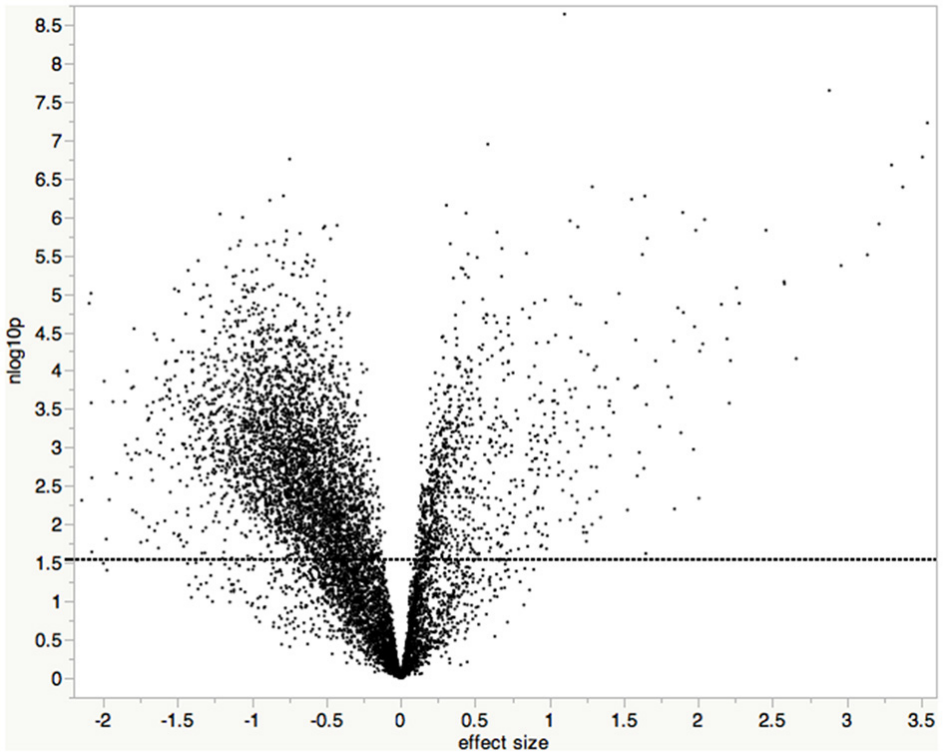
**Volcano plot of test results showing statistical significance vs. fold change**. Each point represents the results of one gene, where the x-axis is the difference in expression between *A. flavus*-infected samples and mock-infected samples (log_2_ [*A. flavus*]—log_2_ [mock]). The y-axis is the –log_10_ transformed *p*-value. The dashed line indicates the significance threshold based on an FDR of 0.05 (Benjamini and Hochberg, 1995); all points above the line are considered statistically significant.

CEL files generated from the GeneChip DNA microarray scans were imported into JMP Genomics and log_2_ transformed. Mismatched probes were not used in the calculation of the expression values. The expression profiles of *A. flavus* and maize genes were examined for each array, and arrays for the mock-inoculated treatment that had moderate-to-strong *A. flavus* signal intensities were removed from further analysis. While these kernels did not visually appear infected, they were likely inadvertently contaminated with *A. flavus*. Data were then normalized using Loess Normalization. Normalized data from arrays generated from blister (R2), milk (R3), dough (R4), and dent (R5) inoculated kernels stages were grouped into either a mock-inoculated or *A. flavus* inoculated treatment group. The assemblage of the different developmental stages into a single treatment group allowed for the identification of maize genes that consistently responded to *A. flavus* infection regardless of what age infection initiated. An analysis of variance (ANOVA) was performed comparing the mock- and *A. flavus*-inoculated treatment groups. To account for multiple testing, a significance threshold based on a false discovery rate (FDR) of 0.05 was used (Benjamini and Hochberg, 1995). The data were deposited into Gene Expression Omnibus. The series record number is GSE57629 (http://www.ncbi.nlm.nih.gov/geo/query/acc.cgi?acc=GSE57629). A volcano plot was generated showing significance on the y-axis and fold change on the x-axis using JMP 11 (Figure 1). Gene names were assigned by using Tophat to align the affy probe sequences to the ZmB73_RefGen_v2 reference genome. AgriGO was used to perform Singular Enrichment Analysis (SEA) on differentially expressed genes (Du et al., 2010).

For SEA, the AGRIGO toolkit was used (http://bioinfo.cau.edu.cn/agriGO/analysis.php). Default values were used for the advanced options including the Yekutieli (FDR under dependency) multi-test adjustment method at a significance level of 0.05.

### Validation of microarray data by qRT-PCR

For each of the developmental stages used for the microarray study, a second set of RNA isolations was performed. RNA was treated with DNase (Promega) and cDNA was synthesized using a First Strand cDNA Synthesis Kit (Fermentas). Quantitative real-time RT-PCR (qRT-PCR) was performed using a SYBR® Green kit (Applied Biosystems) according to the manufacturer's instructions. The expression levels of a ribosome gene were used for normalization. Data were analyzed by the comparative CT method with the amount of target given by the calibrator 2^−ΔΔCT^. The primers used for qRT-PCR analysis are listed in Table 1.

**Table 1:** **Primers used in qRT-PCR**.

**Gene annotation (gene name)**	**Primers 5′–3′**
Structural constituent of ribosome (LOC100285698)	Ribosome F: GGCTTGGCTTAAAGGAAGGT
	Ribosome R: TCAGTCCAACTTCCAGAATGG
PRms (Pathogenesis related protein, maize seed) (AC205274.3_FG001)	PRms F: TACAATGGAGGCATCCAACA
	PRms R: CTGTTTTGGGGAGTGAGGTA
β-fructofuranosidase (invertase cell wall1) (GRMZM2G139300)	CWINV1 F: CGGCAAGATCACCCTTAGAA
	CWINV1 R: CGTAGAGGTGAGCGTCCTTC
1,4-alpha-glucan branching enzyme (GRMZM2G088753)	SBE F: TAGCCCTGGACTCTGATGCT
	SBE R: CCGGTTGTTGAAGTTCGTTT
Lipoxygenase4 (GRMZM2G109056)	LOX4 F: ATCGAGATCCTCTCCAAGCA
	LOX4 R: CTGATCCGCTTCTCGATCTC
Lipoxygenase9 (GRMZM2G017616)	LOX9 F: CCTCATGGCATCAGACTCCT
	LOX9 R: GAGCTGCACATACGACTCCA

## Results

### Differentially expressed genes

Maize kernels were inoculated at the blister (R2), milk (R3), dough (R4), and dent (R5) stages, and harvested 4 days later. Transcriptional changes for 8875 maize genes were monitored with an Affymetrix GeneChip® DNA array. Data were grouped into either mock-inoculated or *A. flavus*-inoculated treatment groups. An ANOVA comparing mock-inoculated with *A. flavus- inoculated* treatment groups (α ≤ 0.05 FDR) identified 912 and 3737 of the Affymetrix GeneChip probe-sets up- and down-regulated, respectively, (Table S1). Each probe-set represents a unique maize gene, except for those with a suffix attached to the probe-set name (e.g., ZM_a_at). These probe-sets may contain probes that represent more than one gene within a gene family or contains conserved sequence common to multiple genes. Some maize genes are represented by multiple probe-sets. Consult http://www.affymetrix.com/estore/support/help/faqs/mouse_430/faq_8.jsp for more detail on the different suffixes. This analysis showed the differential expression of genes associated with host resistance and defense signaling pathways, and of genes associated with sugar metabolism.

### Gene enrichment analysis (SEA)

To gain additional insight into the collective biological function of proteins whose genes showed differential expression during infection, we performed annotation enrichment using SEA (Du et al., 2010) on the genes listed in Table S1. The resulting list of enriched Gene Ontology terms is shown in Table S2. Notable are transcriptional changes in several genes associated with carbohydrate metabolism. Representative examples include GO:0005975, GO:0006006, GO:0034637, GO:0044262, and GO:0019318.

### Changes in expression of genes associated with carbohydrate metabolism

**Figure 2:**
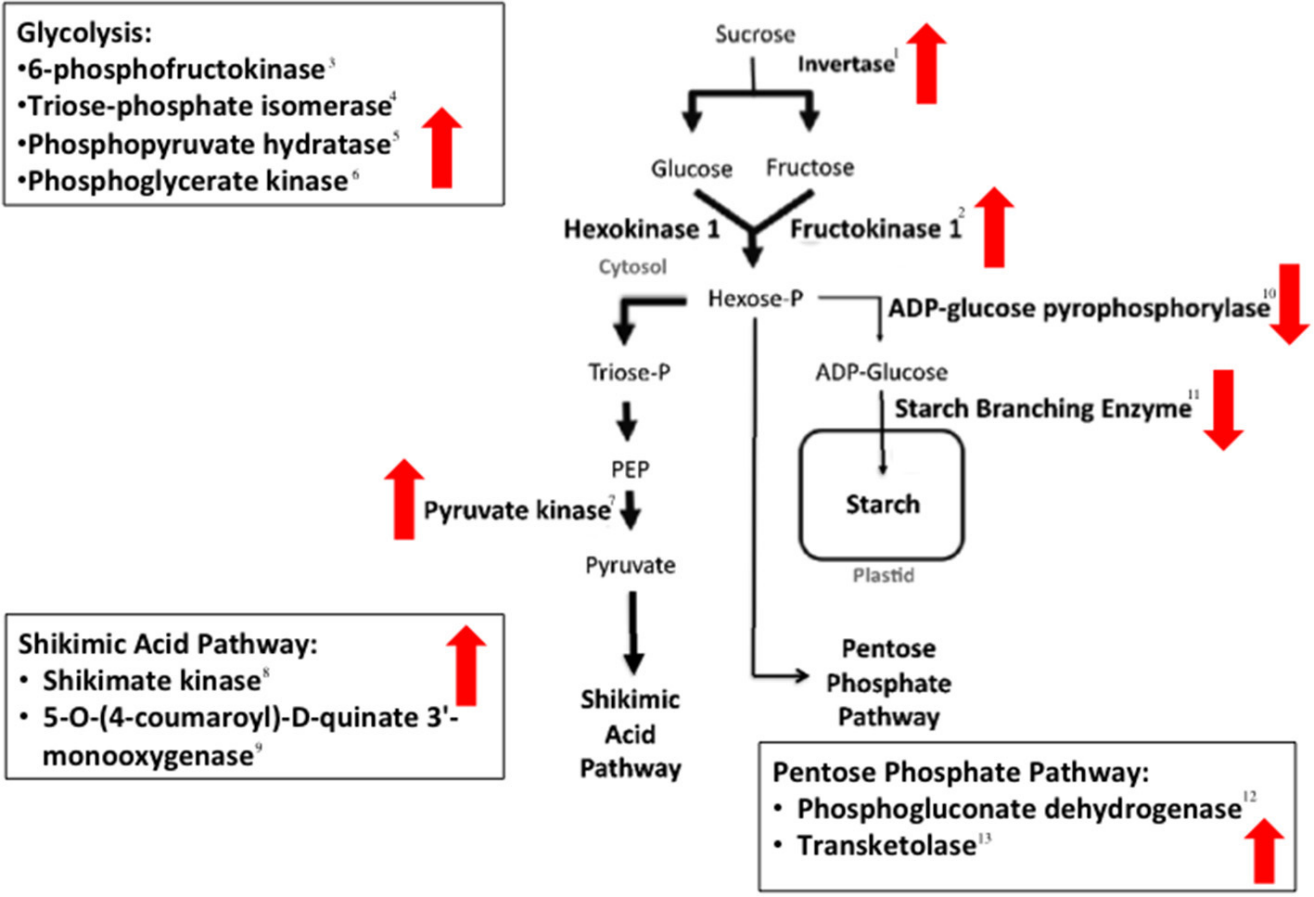
**Enzymes in maize seed carbohydrate metabolism whose biosynthetic genes are differentially expressed in the *A. flavus*-maize interaction**. Black arrows denote described carbohydrate metabolic pathways. Up or down directed block arrows indicate increased or decreased expression of genes, respectively. Numbers beside the enzymes corresponds to their description in Table 2.

Infection of maize kernels with *A. flavus* resulted in transcriptional changes of several maize genes involved in primary and secondary metabolism, particularly those associated with the synthesis and hydrolysis of starch, and the mobilization of hexoses (Figure 2; Table 2; Table S2). As an example, genes encoding starch biosynthetic enzymes, including two starch branching enzymes, (GRMZM2G088753 and GRMZM2G032628), were down regulated during infection as was ADP-glucose pyrophosphorlase (GRMZM2G429899), which catalyzes a key metabolic step in the synthesis of starch in higher plants (Greene and Hannah, 1998). In addition to the apparent down regulation of starch synthesis, there was an increase in transcription of genes involved in starch hydrolysis. The transcription of a β-amylase-like genes, GRMZM2G025833, was upregulated during *A. flavus* pathogenesis.

**Table 2:** **Statistically significant differentially expressed genes referenced in Figure 2**.

Figure 2 References	**Probe ID**	**Gene name**	**Putative protein**	**Fold change**
1	TC302492_ZM_at	GRMZM2G394450	Sucrose:sucrose fructosyltransferase (invertase1)	2.3
1	TC281577_ZM_s_at	GRMZM2G139300	β-fructofuranosidase (invertase cell wall1)	6.0
1	TC309652_ZM_at	GRMZM2G123633	β-fructofuranosidase (invertase cell wall3)	1.3
2	TC292194_ZM_at	GRMZM2G08037	6-phosphofructokinas	2.2
3	TC292194_ZM_at	GRMZM2G080375	6-phosphofructokinase	1.7
4	TC279919_ZM_x_at	GRMZM5G852968	Triose-phosphate isomerase	1.3
5	TC310338_ZM_x_at	GRMZM2G046679	Phosphopyruvate hydratase	1.4
6	TC289234_ZM_at	GRMZM2G051806	Phosphoglycerate kinase	1.8
7	TC285970_ZM_s_at	GRMZM2G150098	Pyruvate kinase	2.1
8	TC311287_ZM_at	GRMZM2G161566	Shikimate kinase	1.6
9	TC299754_ZM_at	GRMZM2G138074	5-O-(4-coumaroyl)-D-quinate 3'-monooxygenase	3.2
10	TC310488_ZM_at	GRMZM2G429899	Glucose-1-phosphate adenylyltransferase (Sh2)	−1.6
11	TC311334_ZM_at	GRMZM2G088753	1,4-alpha-glucan branching enzyme	−3.5
12	TC311531_ZM_s_at	GRMZM2G127798	Phosphogluconate dehydrogenase (decarboxylating)	1.5
13	TC305088_ZM_at	GRMZM2G033208	Transketolase	2.0

Associated with changes in starch accumulation were changes in the mobilization of hexoses. Three maize invertases (GRMZM2G139300, GRMZM2G394450, GRMZM2G123633) were more highly expressed in the *A. flavus* infected kernels than in non-infected kernels (Table 2; Figure 2). Invertases are responsible for hydrolyzing sucrose into glucose and fructose (Cheng et al., 1996; Chourey et al., 2006), and are important in maize kernel development (Weber et al., 1997; Roitsch et al., 2003). Up regulation of invertases in the maize kernel is predicted to cause an increase in free hexose levels in the kernel and affect seed storage reserves such as starch.

The conversion of sucrose to hexoses also was associated with the down regulation of six genes involved with starch biosynthesis. Genes in the starch biosynthetic pathway [*wx1* (GRMZM2G024993), *su1* (GRMZM2G138060), *ss1* (GRMZM2G129451), *sbe1* (GRMZM2G088753), *su2* (GRMZM2G348551), and *ae1* (GRMZM2G032628)] as well as over 20 zein-annotated genes (Table S1) and the gene encoding the transcription factor that regulates 22-kD zein expression, *opaque2* (GRMZM2G015534), were all down-regulated during infection.

It was not possible to determine the exact pathways of hexose remobilization in our studies, but gene expression in both the glycolytic pathway and the Pentose Phosphate Pathway (PPP) was altered by infection (Figure 2; Table 2). Before glucose can be utilized by either pathway, it must be phosphorylated by a hexokinase (Figure 2; Spielbauer et al., 2006). The fructokinase (GRMZM2G080375) was up regulated during pathogenesis. Hexose kinases have been associated with sugar sensing in plants and are potentially involved in the plant defense response (Granot et al., 2013). Glucose-6-phosphate also can be involved in starch synthesis, but because starch biosynthesis genes are down regulated it is likely used by other pathways for the production of energy or defense-related compounds during pathogenesis.

Up regulation of genes in the shikimate pathway supports the premise that hexoses are shunted away from starch synthesis in *A. flavus* infected kernels (Figure 2; Table 2). Several bioreactive compounds from this pathway are known to be involved in host defense (Daayf and Lattanzio, 2009). The shikimate pathway is an entry to aromatic secondary metabolism (Herrmann, 1995) and chorismate synthesized from this pathway is used to make the aromatic amino acids Phe, Tyr, and Trp. These amino acids are precursors for aromatic secondary metabolites including flavonoids and phytoalexins (Herrmann, 1995). Genes in the flavonoid pathway, *fht1* [(GRMZM2G062396), *c2* (GRMZM2G422750) (Bruce et al., 2000)] and genes from other phenypropanoid pathways (Table S1) increased in transcription after *A. flavus* inoculation.

The shikimic acid pathway also provides precursors for the biosynthesis of lignins (Herrmann, 1995), compounds associated with basal resistance to pathogens. Genes involved in lignin biosynthesis have been reported to be induced after *A. flavus* infection in both susceptible (VA35) (Kelley et al., 2012) and resistant varieties (Eyl25) (Luo et al., 2011) of maize. Liang et al. (2006) found lignin concentrations to increase in response to infection by *A. flavus*, and they found a negative correlation between lignin content of peanut cultivars and infection by *A. flavus*. Magbanua et al. (2013) following colonization by a GFP expressing strain of *A. flavus*, found less colonization of maize cob tissue in the resistant inbred Mp313e than in cobs of SC212m, a more susceptible genotype. They attributed the more restricted growth in Mp313e to the highly cross-linked lignin found in Mp313e. In our study, infection of maize kernels by *A. flavus* led to higher expression of three genes (GRMZM2G099420, GRMZM2G131205, GRMZM2G090980) involved in lignin biosynthesis (Table S1).

The carbohydrate metabolic methylerythritol phosphate (MEP) pathway was also found differentially expressed during infection. The following genes from this pathway, which utilizes pyruvate from glycolysis to produces an assortment of isoprenoids including the hormone abscisic acid (ABA) were likewise up-regulated during *A. flavus* infection: (GRMZM2G056975, GRMZM2G493395, GRMZM2G172032, GRMZM2G027059, GRMZM5G859195). We found one gene, 9-cis-epoxycarotenoid dioxygenase (GRMZM2G014392), involved in ABA synthesis up-regulated. The MEP pathway expression has been found induced in maize root colonized by arbuscular mycorrhizal fungi (Lange et al., 2000; Walter et al., 2000).

### Defense signaling pathways

Phytohormones are chemical compounds synthesized by the plant that regulate biochemical processes necessary for growth, reproduction, and survival. The plant defense response is hormonally regulated predominantly by the phytohoromones salicylic acid (SA), jasmonic acid (JA), and ethylene (ET) (Niu et al., 2011; Mengiste, 2012; Derksen et al., 2013). Each hormone likely activates different components of the defense response system that are effective against specific pathogens. The JA/ET pathways are often induced in resistance to necrotrophic pathogens, whereas the SA pathway is typically induced by biotrophic and hemibiotrophic pathogens (Glazebrook, 2005; Derksen et al., 2013).

In this study kernel infection by *A. flavus* resulted in increased expression of the 12-oxo-phytodienoic acid reductases (OPR) encoding *ZmOPR3* (GRMZM2G000236), and the alcohol dehydrogenase encoding *ts2* (GRMZM5G840653). Expression of these genes has been linked with JA biosynthesis in maize (Vick and Zimmerman, 1984; Browse, 2009), and *Ts2* has been associated with the hypersensitive response and resistance to Northern Leaf Blight in maize (Delong et al., 1993; Wisser et al., 2011). Our finding suggests that JA may be involved in the kernel-*A. flavus* interaction.

Other lipid-derived defense-related compounds besides JA are generated from enzymes generally associated with the JA-biosynthesis pathway. As an example, plants contain multiple LOX and OPR genes. In maize, the exact copy number of functional LOX genes varies between maize genotypes (De La Fuente et al., 2013). The function for most LOX and OPR isoenzymes is independent from JA biosynthesis. LOXs catalyze the formation of various oxidized lipids called oxylipins that can act as signaling molecules separate from JA and are thought to have antimicrobial properties (Blée, 2002; Prost et al., 2005). Oxylipins are known to effect fungal growth, including that of *A. flavus*, and mycotoxin production in seeds (Burow et al., 1997; Wilson et al., 2001; Brodhagen and Keller, 2006). The functional role for most OPR isoenzymes is unknown. *ZmLOX* and *ZmOPR* genes were previously found expressed in response to *A. flavus* and other maize fungal pathogen infections (Wilson et al., 2001; Zhang et al., 2005). In accordance with these findings, we observed *LOX* and *OPR* genes differentially expressed during *A. flavus* kernel colonization. *ZmLOX4*, *7*, and *9* (GRMZM2G109056, GRMZM2G070092, GRMZM2G017616) were up-regulated during *A. flavus* infection, whereas *ZmLOX11* (GRMZM2G009479) was down-regulated. *ZmOPR1*, *2*, *3*, and *5* (GRMZM2G106303, GRMZM2G000236, GRMZM2G156712, GRMZM2G087192) were up-regulated in the diseased kernel with *OPR1* and *OPR3* having a 18 and 16 fold-change, respectively, (Table S1).

Whether JA and the other lipid-derived compounds increase maize resistance against pathogen attack may depend on the pathogen and which isoenzyme is expressed. Disruption of the *ZmLOX3* results in enhanced resistance to *F. verticillioides* (Gao et al., 2009), *Colletotrichum graminicola* (Gao et al., 2007), *Cochliobolus heterostrophus* (Gao et al., 2007), and *Exserohilum pedicellatum* (Isakeit et al., 2007). However, the maize *lox3* mutant shows increased susceptibility to *A. flavus* and *A. nidulans*, indicating this gene regulates disease resistance in a pathogen-specific manner (Gao et al., 2009).

### Defense-associated genes in *A. flavus* infected seeds

Pathogenesis-related (PR) proteins are the hallmark of the induced defense response and their expression has been associated with resistance (van Loon et al., 2006; Luo et al., 2011). Several genes annotated as encoding for PR-proteins including those for chitinases [GRMZM2G112538, GRMZM2G477128, *PR-10* (GRMZM2G075283).

GRMZM2G051943, GRMZM2G129189, GRMZM2G133781, *chn2* (GRMZM2G145461), GRMZM2G145518, GRMZM2G162359] were up-regulated in the *A. flavus* infected kernels (Table S1). The expression of *chitinase2* and *PR-10* genes has been reported to be induced in fungal infected maize seed (Cordero et al., 1994). Furthermore, studies by Chen et al. (2006) showed that PR-10 has antifungal activity against *A. flavus in vitro*, and its production is increased upon *A. flavus* infection in the resistance line GT-MAS: gk, but not in the susceptible line Mo17. They also showed that repression of maize *PR-10* by RNAi gene silencing resulted in increased susceptibility to *A. flavus* and aflatoxin production (Chen et al., 2010). A Bowman-Birk-like proteinase inhibitor (GRMZM2G156632), which encodes a PR-like protein showed a 2.5 fold increase in gene expression during infection (Rohrmeier and Lehle, 1993). This gene has been associated with the maize hypersensitive response (Simmons et al., 2002; Chintamanani et al., 2010).

The oxidative burst is an integral part of early plant immunity and is associated with reactive oxygen species, programmed cell death, and the hypersensitive response (Lamb and Dixon, 1997; Dickman and Fluhr, 2013). This defense cascade leads to the production of antimicrobial compounds. Associated with these defense responses are the production of peroxidases and gluathione-S-transferases (GST). Several genes encoding peroxidase-annotated genes (AC197758.3_FG004GRMZM2G080183, GRMZM2G089959, GRMZM2G095404, GRMZM2G103342, GRMZM2G108207, GRMZM2G138918, GRMZM2G149273, GRMZM2G173195, GRMZM2G320269, GRMZM2G321839, GRMZM2G382379, GRMZM2G419953, GRMZM2G441541, GRMZM2G471357) were up-regulated in the diseased kernels.

Four additional peroxidase-encoding genes (GRMZM2G034896, GRMZM2G089895, GRMZM2G103169, GRMZM2G315176) were down-regulated, implying that only certain peroxidase-isozymes are needed during *A. flavus* infection. Gluathione-S-transferases (GST) reduce host cellular damage by detoxifying toxins and xenobiotics commonly encountered during periods of disease and abiotic stress. Wisser et al. (2011) recently correlated ZmGST23 (NP_001104994.1) with moderate resistance to multiple maize pathogens. Though *ZmGST23* was not differentially expressed in this study, the expression of GRMZM2G01909, predicted to encode a GST, had increased expression during *A. flavus* infection.

### Validation of microarray data by qRT-PCR

In order to validate the results of the microarray study, the expression levels of five selected genes were monitored by qRT-PCR: (AC205274.3_FG001, GRMZM2G139300, GRMZM2G088753, GRMZM2G109056, GRMZM2G017616). The fold changes of these genes as determined by qRT-PCR were highly correlated with the results obtained from the microarrays (Table 3).

**Table 3:** **qRT-PCR results for select differentially expressed genes are consistent with microarray results**.

**Gene name**	**Annotation**	**Fold change using microarray**	**Fold change using qRT-PCR**
AC205274.3_FG001	PRms (Pathogenesis related protein, maize seed)	3.1	8.8
GRMZM2G139300	β-fructofuranosidase (invertase cell wall1)	4.0	8.2
GRMZM2G088753	1,4-alpha-glucan branching enzyme	−3.5	−2.3
GRMZM2G109056	Lipoxygenase4	1.9	4.4
GRMZM2G017616	Lipoxygenase9	1.2	2.1

### Physical changes within kernel in response to natural infection with *A. flavus*

**Figure 3:**
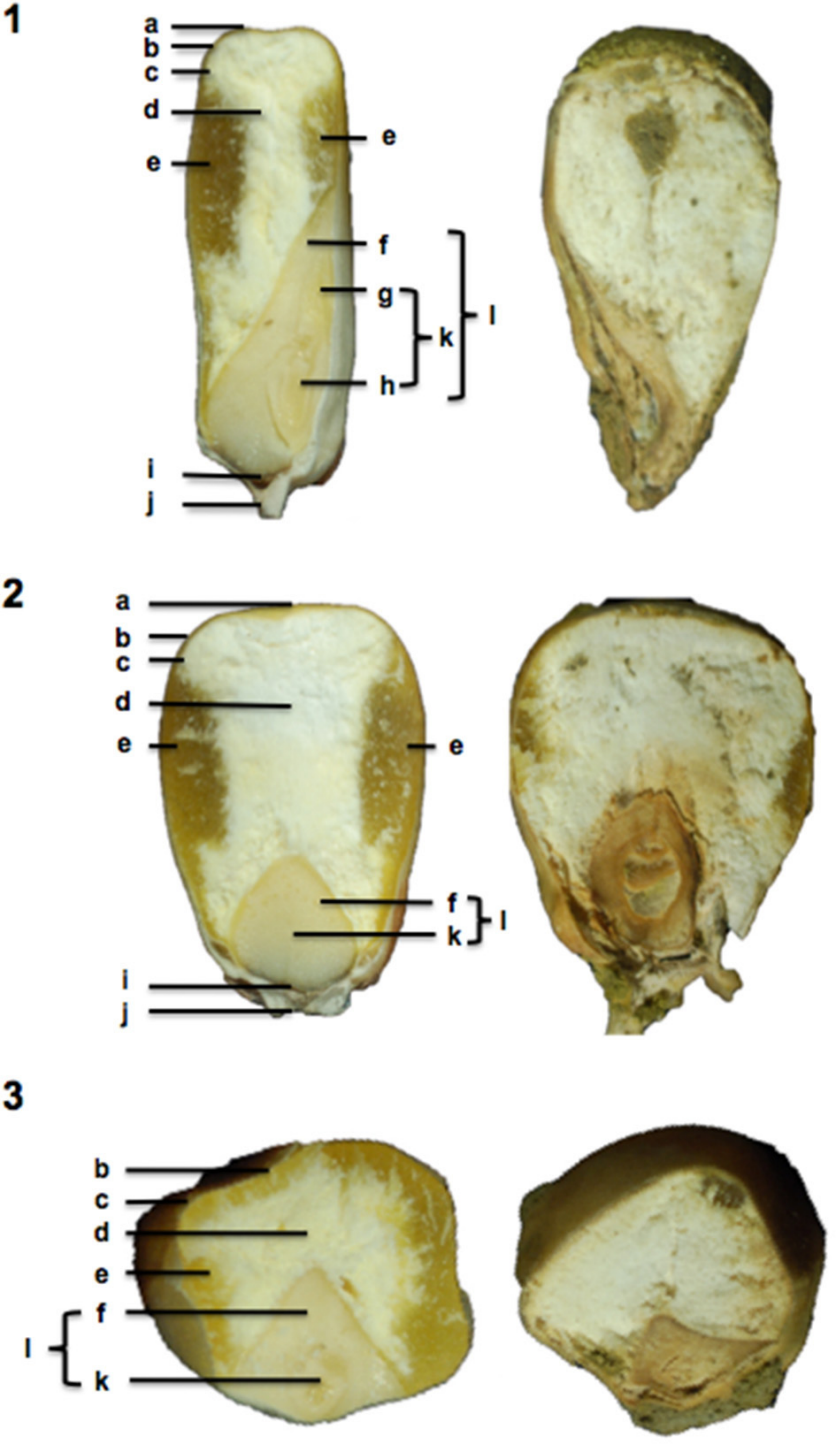
**Mature B73 kernels naturally infected with *Aspergillus flavus***. A sagittal (1) and frontal (2) and transversal (3) section of healthy B73 kernels (left) were compared to disease kernels (right) to discern any physical changes that occurred as a result of *A. flavus*. Bolded letters denote kernel parts and tissues: **a**—crown; **b**—pericarp; **c**—aleurone; **d**—starchy endosperm; **e**—hard endosperm; **f**—scutellar tissue; **g**—leaf primordia (plumule); **h**—primary root; **i**—transfer cells; **j**—pedicel; **k**—embryo; and **l**—germ.

The molecular analysis of maize gene expression during pathogenesis indicated major metabolic effects within kernels in response to infection by *A. flavus*. To determine if such effects could be manifest in the physical structure of kernels, we examined naturally infected kernels at the end of the growing season. Maize kernels adjacent to wound-inoculated kernels were harvested at maturity, dissected, and examined for growth of *A. flavus* and structural integrity. These kernels did not show any obvious wounds or cracks within their pericarp. Figure 3 shows a comparison of three representative infected kernels and non-infected kernels. The most striking modification was the reduced size of the zein-filled hard [horny] endosperm in infected kernels (Figure 3e). In diseased kernels the hard endosperm had been replaced with starchy endosperm (Figure 3d), but the consistency of the entire starchy endosperm was different from that of the non-infected kernel. Instead of being firm and intact, the starchy endosperm of the diseased kernel was fragile, friable, and filled with tiny air pockets. *A. flavus* could be discerned in some of these pockets including the gap between endosperm and germ (Figure 3d, l). Mycelium was also observed in the embryo around the plumule (Figure 3g) and primary root (Figure 3h), and the germ was discolored and shriveled (Figure 3f).

## Discussion

*A. flavus* kernel colonization is most aggressive on maize plants that have been subjected to heat or water stress (Tubajika and Damann, 2001; Scheidegger and Payne, 2003; Widstrom et al., 2003; Guo et al., 2008). Such conditions frequently occur throughout the world on rain-fed fields, and thus *A. flavus* colonization of kernels and subsequent contamination with aflatoxins is a concern internationally. Resistance to aflatoxin accumulation shows low heritability in the field, owing to the quantitative nature of resistance, the lack of reliable phenotyping, and strong genotype by environment (GxE) interactions. Thus, any approach that facilitates the identification of genes contributing to host resistance could accelerate the development of resistant maize genotypes.

The overall goal of this study was to better characterize host response to *A. flavus* opportunistic infection by identify maize genes differentially expressed during infection that could have applications as genetic markers in future breeding programs. Our previous research (Dolezal et al., 2013) showed that *A. flavus* follows a predictable pattern of colonization after inoculation of maize kernels. While all tissue types of the kernel can be colonized, growth of the fungus into scutellum tissue appears to follow the formation of a biofilm-like structure by *A. flavus* (Dolezal et al., 2013). The scutellum is metabolically active and known to synthesize numerous hydrolytic enzymes and defense-associated compounds (Casacuberta et al., 1991, 1992). Based on these studies, we chose 4 days after inoculation as the time to evaluate host response to *A. flavus*. Data presented in this study indicate that infected seed at this time to be transcriptionally responsive and express genes known to be involved in host defense.

Our observations show that B73 kernels mount a multipronged defense response to *A. flavus* typical of that associated with plant basal resistance. Many of the maize genes induced by *A. flavus* are important in resistance against maize foliar pathogens, underscoring a possible commonality of the resistance response in seeds and leaves. These data further suggest that *A. flavus* infection invokes defense tactics used against more aggressive maize pathogens including increased transcription of defense signaling pathways and genes several genes known to be involved in the host's defense response.

We also observed striking changes in the transcription of genes associated with carbohydrate utilization (Table S2). An analysis of these transcriptional changes leads us to conclude that infection by *A. flavus* decreases starch synthesis, increases starch degradation, and mobilizes hexoses into pathways associated with plant defense (Figure 2; Table 2). A physical examination of kernels naturally infected with *A. flavus* (Figure 3) showed changes in the structure of the maize endosperm that could reflect the remobilization of hexoses in the seed in response to infection.

Physical changes in seeds infected with fungi have been observed before (Fennell et al., 1973; Koltun et al., 1974; Huff, 1980; Shetty and Bhat, 1999; Cardwell et al., 2000; Pearson and Wicklow, 2006). Most researchers have speculated that hydrolytic enzymes secreted by infecting fungi are responsible for this loss in grain quality. However, maize mutants with abnormal expression levels of carbohydrate and protein biosynthetic pathway genes can also develop atypical endosperm tissue (Neuffer et al., 1997; Black et al., 2006). Our findings showing changes in kernel primary metabolism during *A. flavus* infection challenges the assumption that fungal produced enzymes are solely responsible for changes in kernel structure, and suggests the plant may also contribute to these changes through starch degradation and hexose mobilization away from starch synthesis.

While these metabolic changes could represent a defense response by the kernel to infection by *A. flavus*, the changes could instead promote host susceptibility to the pathogen. Fungi, particularly fungal plant pathogens, are capable of manipulating the plant's metabolism to create an environment advantageous for fungal growth (Govrin and Levine, 2000; Doehlemann et al., 2008). Increased invertase transcription in *A. flavus* infected kernels could indicate a higher-than-normal accumulation of free-hexoses within diseased tissue. Because glucose is the preferred carbon source of *A. flavus*, the up-regulation of sucrose hydrolyzing enzymes would presumable promote disease development by providing a steady supply of nutrients to the pathogen. IVR1 was previously found induced in sugar-poor environments, and its expression associated with tumor formation in *Ustilago maydis* infected maize (Xu et al., 1996; Doehlemann et al., 2008). Simple carbohydrates are also known to promote aflatoxin in maize kernels. Woloshuk et al. (1997) found an *A. flavus* α-amylase to play an important role in the production of aflatoxin by providing simple sugars conducive for aflatoxin production. Thus, sugar status in kernels could condition increased susceptibility as well as aflatoxin contamination.

In contrast, other studies have noted increased levels of hexoses in-and-around the site of pathogen infection and have hypothesized that these starch-derived sugars are an integral component of the host defense response (Berger et al., 2007; Bolton, 2009). Free-hexoses are thought to be used in the generation of reducing agents [NAD(P)H], energy [ATP], and pathway intermediates needed to synthesize secondary metabolite compounds. Their presence may also help trigger the synthesis of defense-related compounds.

Data from these studies, along with previous transcriptional studies (Luo et al., 2009, 2011; Jiang et al., 2011; Kelley et al., 2012), lay the groundwork for future studies investigating *A. flavus* resistance in maize. Under normal growth conditions, inducible defenses of B73 genotype may be adequate in inhibiting or at least slowing down *A. flavus* disease development. However, external and internal factors could affect this response. Abiotic stress, such as drought, can have a negative impact on the defense response (Wotton and Strange, 1987; Duke and Doehlert, 1996; Luo et al., 2010). Also, inherently low expression of defense and defense-associated genes may predispose the plant to greater infection (Chen et al., 2001; Alessandra et al., 2010). Genes expressed during infection may not necessarily be involved in resistance and could be causing increased susceptibility to fungal disease. Knowing which genes are typically expressed in response to pathogen attack is useful when examining how genotype and abiotic stress influence the infection process. Progress on more fully understanding disease development will ultimately leads to the development of genetically resistant cultivars.

### Conflict of interest statement

The authors declare that the research was conducted in the absence of any commercial or financial relationships that could be construed as a potential conflict of interest.
